# Development and validation of the LoVI: the Laws on Violence against women and girls Index

**DOI:** 10.1186/s12914-020-00233-z

**Published:** 2020-05-29

**Authors:** Kathryn M. Yount, Patricia C. Lewis, Cari Jo Clark, Lori Heise, Ruchira T. Naved, Lauren Maxwell

**Affiliations:** 1 Hubert Department of Global Health, Emory University, 1518 Clifton Rd, NE, Atlanta, GA 30322 USA; 2 Department of Sociology, Emory University, 1555 Dickey Dr, Atlanta, GA 30322 USA; 3 Department of Population, Family, and Reproductive Health, Johns Hopkins Bloomberg School of Public Health and JHU School of Nursing, 615 N. Wolfe Street., Baltimore, Maryland 21205 USA; 4 International Centre for Diarrheal Disease Research, 68 Shaheed Tajuddin Ahmed Sarani Mohakhali, Dhaka, 1212 Bangladesh

**Keywords:** Factor analysis, Gender equality, National Legislation, Sustainable development goals, Index validation, Violence against women and girls (VAWG)

## Abstract

**Background:**

Violence against women and girls (VAWG) is a human-rights violation with adverse long-term and inter-generational consequences. Redefining VAWG as legally unacceptable is one strategy for social change. The co-occurrence of national laws against VAWG is understudied, and tools to monitor the national legal environment are lacking. We developed the Laws on Violence against Women and Girls Index (LoVI) to measure global progress to develop comprehensive national legislation against child marriage, sexual harassment, domestic violence, and marital rape.

**Methods:**

Using data from 2016 and 2018 for 189 countries from the World Bank Women, Business, and the Law database, we used factor analysis to assess the dimensionality of the LoVI. We examined the distribution of the LoVI across countries and regions, and the relationship of national rankings on the LoVI with those for other indicators from the United Nations, Demographic and Health Surveys, and World Factbook.

**Results:**

A single LoVI factor showed good model fit in the factor analysis. National LoVI rankings were positively associated with gender equality in human development and economic rights-related rankings and negatively associated with rates of justifying wife beating and of lifetime and prior-year physical and/or sexual IPV. The LoVI was not associated with national indicators for human development and income inequality.

**Conclusion:**

The LoVI is a concise, coherent, validated index to monitor the progress of nations on adopting comprehensive legislation to advance 2030 Sustainable Development Goal 5, to eliminate VAWG.

## Background

The United Nations (UN) defines violence against women (VAW) as “any act of gender-based violence that results in, or is likely to result in, physical, sexual, or psychological harm or suffering to women, including threats of such acts, coercion or arbitrary deprivation of liberty, whether occurring in public or in private” [1]. VAW and girls (VAWG) includes, but is not limited to, harmful practices, such as child marriage, as well as sexual harassment, intimate partner violence (IPV), and marital rape [1]. VAWG threatens the social, economic, and health-related wellbeing of survivors, exposed children, and societies [2–10]. Advocacy for laws that redefine VAWG as unacceptable has been one strategy for prevention and response. In 2015, the UN embedded in Sustainable Development Goal 5 (SDG5) three ambitious targets, to end “all forms of discrimination against all women and girls everywhere” (5.1); “all forms of violence against all women and girls in the public and private spheres …” (5.2); and “all harmful practices, such as child, early and forced marriage …” (5.3, p.18) [11].

Laws on VAWG may facilitate societal change through multiple pathways, such as enabling citizens to hold states and perpetrators accountable for VAWG [12], attracting resources for primary and secondary prevention [13], and changing societal norms about the treatment of women [14]. A concise index capturing laws on VAWG is needed to monitor legal change more comprehensively and influences of the legal environment on health and economic outcomes. We used the World Bank Women, Business, and the Law (WB-WBL) database to create and validate the Laws on Violence against Women and Girls Index, or LoVI. The LoVI measures the comprehensiveness of national anti-violence legislation with respect to four types of VAWG: child marriage, sexual harassment, IPV, and marital rape. These forms of violence were selected because they exist to varying degrees in all countries and capture risks experienced disproportionately by women and girls. These forms of violence also occur at different life stages (childhood and adulthood), in different venues (home, work, and school) and in different types of relationships (intimate partnerships, marriage, and colleague/peer relations). Legislation about these forms of violence also capture salient expressions of State opposition to historical forms of male entitlement involving access to, control over, and even ownership of women’s bodies.

Although policy analyses tend to assess the effects of a single law on a specific outcome, in reality, laws exist in clusters. These clusters establish legal norms or expectations about the treatment of women. By applying factor analysis to create the LoVI from publicly available, longitudinal data on existing laws, we capture a more nuanced measure of the legal context with respect to VAWG. The creation of an index to provide an objective measure of the comprehensiveness of national laws against VAWG is supported by theory on the social ecology of VAWG [15], which identifies macro-level factors such as laws and societal norms as influences on the risk of VAWG. The creation of an index also is supported by evidence on the co-occurrence of multiple types of VAWG [16], such as child marriage and intimate partner violence [17].

We developed and evaluated the LoVI in four steps. First, we created and validated the LoVI to measure comprehensive national legislation with respect to four types of VAWG. Second, we ranked countries, regions, and income levels on the LoVI to understand how legal environments with respect to VAWG vary worldwide in 2018. Third, we assessed the concurrent validity of the LoVI vis-à-vis other national indicators for societal norms on VAWG, prevalence of VAWG, violence prevention and response programs, laws on women’s economic equality, overall and gender-related human development, and income inequality. Lastly, we clarified how to compute the LoVI manually for monitoring the progress of nations toward SDG5, to eliminate VAWG.

## Methods

### Data

Data for 189 countries were used to construct the LoVI, and data for between 45 and 189 countries, depending on the indicator, were used to assess concurrent validity, comparing country rankings on the LoVI with country rankings on other national indicators. Table 1 lists data sources, time periods, and sample sizes for each measure.

**Table 1:** Data sources, sample sizes, years, and number of items for composite indicators of the LoVI and other national indicators

Construct and Indicators or Index	Data Source	# of Countries	Years	# of Items (Scale)
***Laws on VAWG Index (LoVI)***				
Sexual harassment	WB-WBL	189	2016/2018	3 (0,1)
Child marriage	WB-WBL	186	2016/2018	1 (0,1)
Domestic violence	WB-WBL	189	2016/2018	5 (0,1)
Marital rape	WB-WBL	189	2016/2018	1 (0,1)
***Laws on Women’s Economic Equality (LoWEE)***	WB-WBL	189	2018	5 (0,1)
***VAWG Programs***				
VAWG Prevention Programs Index (VPPI)	2014 UN Report	133	2014	6 (0,1,2)
VAWG Response Programs Index (VRPI)	2014 UN Report	133	2014	3 (0,1,2) 2 (0,1,2,3)
***Societal Norms about VAWG***				
Justification of physical IPV	DHS, MICS	78	2001–2017	
Exposure to physical/sexual IPV, ever	DHS	45	2005–2017	
Exposure to physical/sexual IPV, in prior year	DHS	46	2005–2017	
**Human development, income inequality**				
Human Development Index (HDI)	Human Development Reports	183	2015	
Gender-related Development Index (GDI)	Human Development Reports	158	2015	
Gini coefficient (Gini)	The World Factbook	157	2003–2016	

*DHS* Demographic and Health Survey, *IPV* intimate partner violence, *LoVI* laws on violence against women and girls index, *MICS* Multiple Indicator Cluster Survey, *UN* United Nations*, VAWG* violence against women and girls, *WB-WBL* World Bank, Women Business and Law database

### National Laws on VAWG

#### LoVI

The LoVI was created from four composite indicators that captured the comprehensiveness of national legislation against child marriage, sexual harassment, domestic (or intimate partner) violence, and marital rape. ‘Comprehensive’ referred to the presence of legislation against each type of VAWG, including definitions of the violent acts covered. To create each composite indicator, we used the same items from the WB-WBL database for 2016 and 2018, allowing for separate analyses of two country samples (Table 1) [18, 19] and for use of the LoVI to monitor legal change over time from an existing, longitudinal data source. *Legislation against child marriage* captured whether the minimum legal age of marriage was 18 years or older (1 if yes for 1 item, 0 otherwise). *Legislation against sexual harassment* captured the presence of legislation against sexual harassment that defined sexual harassment in education and in employment (1 if yes for 3 items, 0 otherwise). *Legislation against domestic violence* captured the presence of legislation against domestic violence and for which acts of economic, emotional, physical, and sexual domestic violence were covered (1 if yes for 5 items, 0 otherwise). *Legislation against marital rape* captured the presence of legislation that explicitly criminalized marital rape (1 if yes for 1 item, 0 otherwise). Detailed definitions of all LoVI items are provided in Supplemental Table S1.

### Other National-Level Indicators

#### Laws on women’s economic equality

The study team created the Laws on Women’s Economic Equality (LoWEE) index from a confirmatory factor analysis (CFA) of five binary items from the 2018 WB-WBL database [19]. Items captured the presence (=1) or absence (=0) of national laws that mandated *non-discrimination on the basis of gender in hiring and employment*, *equal remuneration for work of equal value*, *equal rights of sons and daughters and surviving spouses to inherit assets*, and *non-discrimination on the basis of gender in access to credit*. We assessed CFA model fit based on theory about salient legal aspects of women’s economic equality/empowerment [20], parameter estimates (standardized loadings > 0.35), and model fit statistics that are more robust to small sample size (comparative fit index, CFI, *around* 0.95 or higher) [21–23]. Based on these criteria, the CFA model had reasonable fit: a broad spectrum of economic equality laws were covered, standardized loadings were large (0.68–0.85), and the CFI was 0.92. The final index was rescaled to have a mean of 0.5 and range 0.00–1.00 (actual range 0.05–0.84, results available on request).

#### VAWG prevention and response programs

The *VAWG Prevention Programs Index* (VPPI) was created from a CFA of six ordinal items from the 2014 *Global Status Report on Violence* [24]. Items captured the scale (0 = none, 1 = limited, 2 = large scale) of dating violence prevention in schools, microfinance and gender equity training, socio-cultural norms change related to IPV, sexual violence prevention in schools or colleges, changes to the physical environment to prevent VAWG, and socio-cultural norms change related to sexual violence. Based on theories of violence prevention [13, 16], as well as standardized loadings (0.44–0.90) and model fit (CFI = 0.97), the CFA model had adequate fit. The *VAWG Response Programs Index* (VRPI) was created from a CFA of five ordinal items from the *Global Status Report*. Three items captured the scale (0 = none, 1 = limited, 2 = large scale) of health-provider identification and referral of IPV or sexual-violence survivors, medico-legal services for sexual-violence survivors, and prenatal screening for IPV risk. Two items captured the extent (0 = none, 1 = limited, 2 = partial, 3 = full implementation) of services related to victim compensation from the state and legal representation. Using the same criteria to assess model fit, the CFA model had adequate fit (standardized loadings 0.31–0.92, CFI = 0.94).^1^

#### National normative expectations and prevalence of VAWG

The Demographic and Health Surveys (DHS) [25] STATScompiler (https://www.statcompiler.com/en/) and Multiple Indicator Cluster Surveys (MICS) [26] MICScompiler (http://www.micscompiler.org/) provided national data for 78 countries in 2001–2017 on the percentage of women 15–49 years who justified physical IPV against women in specific situations (a measure of injunctive societal norms). We used the DHS STATcompiler to derive national estimates for 45 countries in years 2005–2017 for the percentages of ever- or currently partnered women 15–49 years who experienced lifetime physical and/or sexual IPV, and who experienced prior year physical and/or sexual IPV (measures of descriptive behavior).

#### Human development, gender-related human development, and income inequality

The *Human Development Index* (HDI) captures a country’s average achievement with respect to its population’s longevity, education, and living standard. The HDI is the geometric mean of the population’s life expectancy at birth, mean grades of schooling for adults 25 years or older and expected grades of schooling for children of school-entering age, and logarithm of gross national income per capita, scaled from 0 to 1 [27]. The *Gender-related Development Index* (GDI) captures a country’s gender gap in human development [27]. The GDI is the ratio of the HDI calculated for women versus that calculated for men; the GDI ranges from 0 to 2 [27]. The *Gini coefficient* measures the deviation of the distribution of income among individuals or households within a country from a perfectly equal distribution [28, 29], with 0 representing absolute equality and 100 representing absolute inequality. These data are publicly available from the cited sources.

### Analysis

All data analyses were performed in MPlus version 8 (Muthén & Muthén, Los Angeles, CA.), Stata SE version 15.1 (StataCorp LP, College Station Texas), and ArcMap version 10.5.1 (Esri, Redlands California). The shapefile used to create the choropleth map of LoVI quintiles was downloaded from www.naturalearth.com on 8 June 2018.

#### Factor analysis

To validate the LoVI, we first conducted univariate analyses to assess the distributions and any missingness of all original WB-WBL items and the four composite indicators. In 2016, three countries had missing data on legal age at marriage for girls (Lebanon, Saudi Arabia, Republic of Yemen). In 2018, 19 countries had missing data on the legal age at marriage for girls. For 16 of these 19 countries, missing data were imputed using the status of the law in 2016, resulting in three countries with missing data in 2018 (Lebanon, Saudi Arabia, Republic of Yemen). In the factor analysis, the three missing observations for child marriage were assumed missing at random. We then estimated pairwise tetrachoric correlations of the composite indicators. These correlations were zero-adjusted such that, when a cell count was zero, the frequency was increased from zero to one-half while maintaining row and column totals. This strategy is recommended for continuity corrections in small samples [30, 31]. Third, we performed exploratory factor analysis (EFA) of the composite indicators from the 2016 WBL database and then CFA of the composite indicators from the 2018 WBL database. For factor analyses (of the LoVI and all indices), we used diagonally-weighted least-squares estimation (WLSMV) with geomin (oblique) rotation, which yields more accurate factor loadings when input variables are ordinal and sample size is small [32]. Again, we assessed model fit based on theory, factor loadings (> 0.35), and a model-fit index that is more robust in small samples (CFI *around* 0.95 or higher) [21–23]. This process allowed us to explore and then to confirm the factor structure of the LoVI in the same countries with the same measures from the same database for two distinct years.

#### National rankings on the LoV

From the CFA model for 2018, we generated factor scores for the LoVI and rescaled the scores to have a mean of 0.50 and range of 0.00 to 1.00. We then ranked countries according to quintiles on the LoVI for 2018 and provided scores and rankings for each country, region, and income level (Supplemental Tables S3 & S4).

#### Comparing the LoVI with other national indicators

As a final step, we used scatterplots with locally weighted regression (LOWESS) to compare country rankings on the LoVI with country rankings on other national indicators. We applied LOWESS to avoid making assumptions about the nature of the relation between the LoVI and other national indicators. We compared national rankings on all measures to address differences in measurement scales across national indicators. Tied values were assigned the same rank while preserving the sum of the ranks. Because hypothesis testing and correlation matrices have been criticized as methods to evaluate the level of agreement between comparable measurements [33, 34], we used Bland-Altman plots [33] to assess visually the relation between country rankings on the LoVI and country rankings on other national indicators. Bland-Altman plots depict the difference between measurements and their mean to quantify the level of agreement between comparable methods of measurement. In each plot, we noted outliers, countries whose rankings on the LoVI and the alternative measures were outside of the 95% limits of agreement.

## Results

### National Laws on VAWG and other National Indicators

In 2016 and 2018, 94% of countries (*n* = 174 of 186) had a legal age of marriage for girls at 18 years or older (Appendix Table S2). In 2016, 29% of countries (*n* = 54) had comprehensive legislation on sexual harassment; in 2018, 34% of countries (*n* = 64) had such legislation. In 2016, 43% of countries (*n* = 82) had comprehensive legislation on domestic violence (Appendix Table S2). By 2018, 49% of countries (*n* = 93) had such legislation. Marital rape was criminalized in 40% of countries (*n* = 76) in 2016 and in 41% of countries (*n* = 78) in 2018.

In 2018, about half of 189 countries (*n* = 95) had laws mandating nondiscrimination based on gender in employment and hiring, and 40% (*n* = 76) had laws mandating equal remuneration for week of equal work (Appendix Table S2). Over three fourths of countries (*n* = 146) gave sons and daughters equal rights to inherit assets from parents, and 80% (*n* = 147) gave surviving female and male spouses equal rights to inherit assets. In 38% of countries (*n* = 72), the law prohibited discrimination on the basis of sex or gender in access to credit.

In 2014 across some 130 countries, between one fifth and two thirds of countries were implementing various VAWG prevention or response programs ‘on a large scale’ (Appendix Table S2). In general, large-scale prevention programming was less prevalent than large-scale response programming. Regarding the former, only about half of countries were implementing socio-cultural norms change programs related to sexual violence (*n* = 67) and IPV (*n* = 65) on a large scale. Only one third (*n* = 48) were implementing sexual violence prevention in schools or colleges on a large scale, and one in five were implementing dating violence prevention (*n* = 29) and microfinance and gender-equity training (*n* = 28) on a large scale. Regarding response programming, two thirds of countries (*n* = 89) were implementing medico-legal services for sexual violence survivors on a large scale, and more than half were implementing health-provider identification and referral of IPV survivors (*n* = 71) and victim representation (*n* = 70) on a large scale. Only two fifths of countries (*n* = 52) were implementing prenatal screening for child maltreatment and IPV risks on a large scale, however.

During 2001–2017 across 78 countries, more than one third of women reported that wife beating was justified. During 2005–2017 across 45 countries, mean lifetime (34.2%, SD = 13.0%) and prior-year (19.0%, SD = 9.4%) physical or sexual IPV were high. The mean HDI in 2015 was 0.7 (SD = 0.2), and the mean GDI in 2015 was 0.9 (SD = 0.1), suggesting that women’s human development still lagged that of men. The mean Gini coefficient in the sample from years 2003–2016 was 38.1 (SD = 8.1), suggesting a high overall level of income inequality.

### Pairwise correlations of National Laws on VAWG

In pairwise tetrachoric correlations of the LoVI items and composite indicators, all but one pair were positively correlated (Table 2). Most correlations were significant at the 0.05 level. All sexual-harassment items were significantly correlated with each other, and all domestic-violence items were significantly correlated with each other. Many of the original sexual-harassment and domestic-violence items (on which the composite indicators were based) were significantly correlated, and the sexual-harassment and domestic-violence composite indicators were significantly correlated in 2018. Child-marriage legislation was significantly correlated with some but not all of the sexual-harassment and domestic-violence items. Marital-rape legislation had the lowest correlations. In 2016 and 2018, marital-rape legislation was not significantly associated with child-marriage legislation, the sexual-harassment composite indicator, or the sexual harassment in education item. Marital-rape legislation had higher, significant correlations with domestic-violence items in both years.

**Table 2:** Pairwise tetrachoric correlations^a^ of national laws against violence against women and girls, using data for 2016 and 2018 from the World Bank’s Women, Business and the Law database

**2016**												
	SH_com	SH_gen	SH_emp	SH_edu	ChildMar	DV_com	DV_gen	DV_phy	DV_sex	DV_emo	DV_eco	MarRape
SH_com	1.00											
SH_gen	0.78*	1.00										
SH_emp	0.88*	0.97*	1.00									
SH_edu	0.99*	0.79*	0.79*	1.00								
ChildMar	0.50*	0.16	0.60*	0.51*	1.00							
DV_com	0.25	0.17	0.23	0.29*	0.38	1.00						
DV_gen	0.42*	0.39*	0.52*	0.43*	0.71*	0.90*	1.00					
DV_phy	0.45*	0.35*	0.50*	0.46*	0.69*	0.91*	0.99*	1.00				
DV_sex	0.31*	0.18	0.34*	0.34*	0.51*	0.98*	0.95*	0.96*	1.00			
DV_emo	0.37*	0.31*	0.49*	0.39*	0.67*	0.92*	0.99*	0.99*	0.97*	1.00		
DV_eco	0.21	0.12	0.17	0.25	0.40*	0.99*	0.91*	0.92*	0.93*	0.93*	1.00	
MarRape	0.24	0.35*	0.45*	0.24	0.35	0.56*	0.64*	0.67*	0.59*	0.65*	0.54*	1.00
**2018**												
	SH_com	SH_gen	SH_emp	SH_edu	ChildMar	DV_com	DV_gen	DV_phy	DV_sex	DV_emo	DV_eco	MarRape
SH_com	1.00											
SH_gen	0.77*	1.00										
SH_emp	0.87*	0.96*	1.00									
SH_edu	0.99*	0.78*	0.79*	1.00								
ChildMar	0.44	−0.02	0.56*	0.45	1.00							
DV_com	0.27*	0.07	0.18	0.30*	0.45*	1.00						
DV_gen	0.52*	0.26	0.44*	0.53*	0.65*	0.91*	1.00					
DV_phy	0.54*	0.23	0.49*	0.55*	0.63*	0.92*	0.99*	1.00				
DV_sex	0.38*	0.09	0.32*	0.40*	0.48*	0.97*	0.97*	0.97*	1.00			
DV_emo	0.49*	0.22	0.43*	0.51*	0.62*	0.92*	0.99*	0.99*	0.97*	1.00		
DV_eco	0.28*	0.09	0.20	0.31*	0.46*	0.99*	0.92*	0.92*	0.95*	0.93*	1.00	
MarRape	0.23	0.26	0.42*	0.23	0.36	0.57*	0.62*	0.64*	0.63*	0.60*	0.57*	1.00

*SH* Sexual Harassment, *_com* Composite, *_gen* general legislation, *_emp* employment, *_edu* education, *ChildMar* Child Marriage, *DV* Domestic Violence, *_phy* physical, *_sex* sexual, *_emo* emotional, *_eco* economic, *MarRape* Marital Rape

Tetrachoric correlations are zero-adjusted so that when a cell has a zero count; Stata increases the frequency from zero to one-half while maintaining row and column totals

*next to figures indicates significance at 0.05 level

### Factor analyses

Exploratory and confirmatory factor models of the four composite legal indicators showed good fit to the data (Table 3). For both models, the CFI exceeded 0.95. All indicators loaded on the factor at or above 0.35. Indicators for legislation against domestic violence and child marriage loaded most strongly, at 0.71 and higher. Legislation against marital rape loaded just slightly lower, at about 0.60, and sexual-harassment legislation loaded at 0.39.

**Table 3:** Exploratory and confirmatory factor analyses of the Laws on Violence against Women and Girls Index (LoVI), using data for 2016 and 2018 from World Bank’s Women, Business, and Law Database

Composite legal indicator	WB 2016 EFA Factor 1 (***N*** = 189)	WB 2018 CFA Factor 1 (***N*** = 189)
Anti-child-marriage legislation	0.71	0.70
Anti-sexual-harassment legislation	0.39	0.39
Anti-domestic-violence legislation	0.74	0.79
Anti-marital-rape legislation	0.58	0.61
**Model fit statistics**		
Comparative Fit Index (CFI)	0.99	1.00

*CFA* confirmatory factor analysis, *EFA* exploratory factor analysis

### Country rankings on the LoVI

**Fig. 1:**
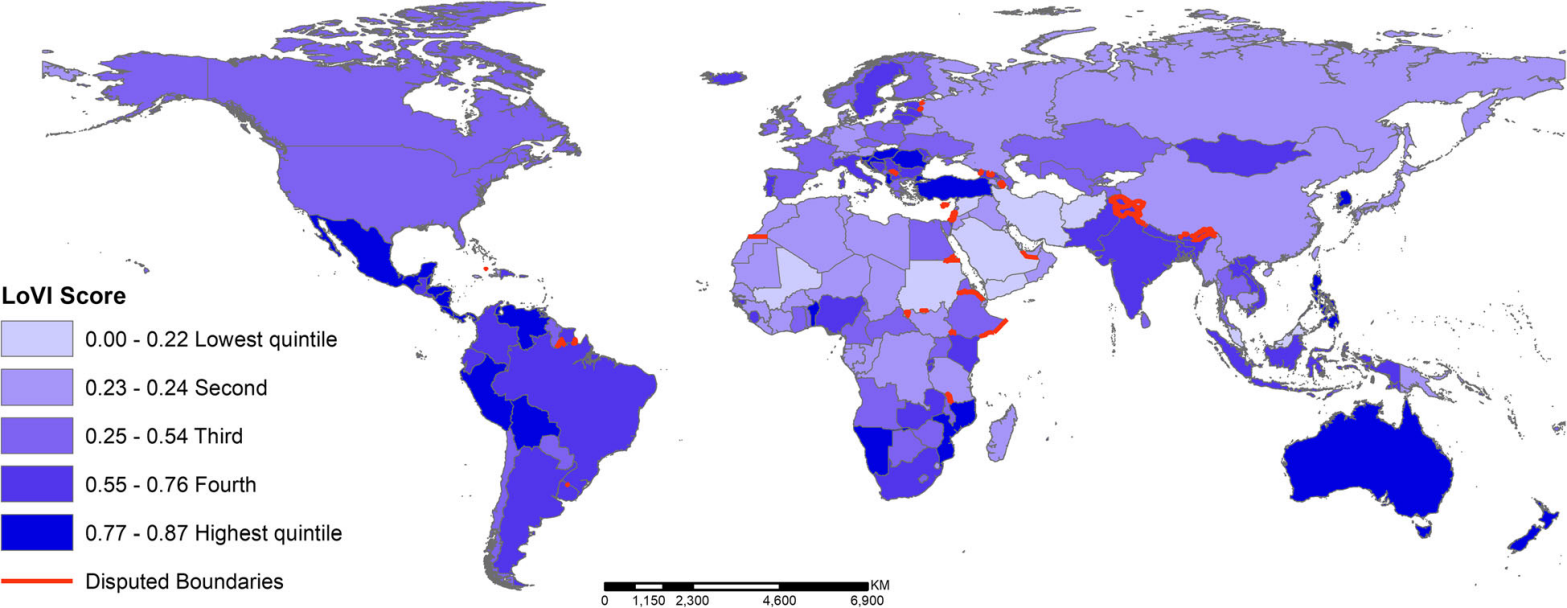
Country rankings by LoVI quintile. Notes. To create the choropleth map of LoVI quintiles, the authors used ArcMap version 10.5.1 (Esri, Redlands California) and the shapefile downloaded from www.naturalearth.com on 8 June 2018. This figure is original, and the author team produced it as part of the analysis for this publication

In Fig. 1, we present a global map of countries by quintiles of the LoVI. In the Americas, Mexico, most of Central America (excluding Guatemala and El Salvador) along with Bolivia, Peru, and Venezuela stood out as having more comprehensive anti-VAWG legislation than other countries in the region. In Africa, Benin, Mozambiqe, and Namibia had the most progressive legal contexts; whereas, Mali and North Sudan were the most restrictive. Some of the most progressive policy contexts were in Eastern Europe, including Croatia, Hungary, Slovenia, Romania, and Turkey. At the regional level, the Middle East had the least and Oceania the most progressive legal contexts with respect to anti-VAWG legislation.

### Comparison of the LoVI with other National Indicators

**Fig. 2:**
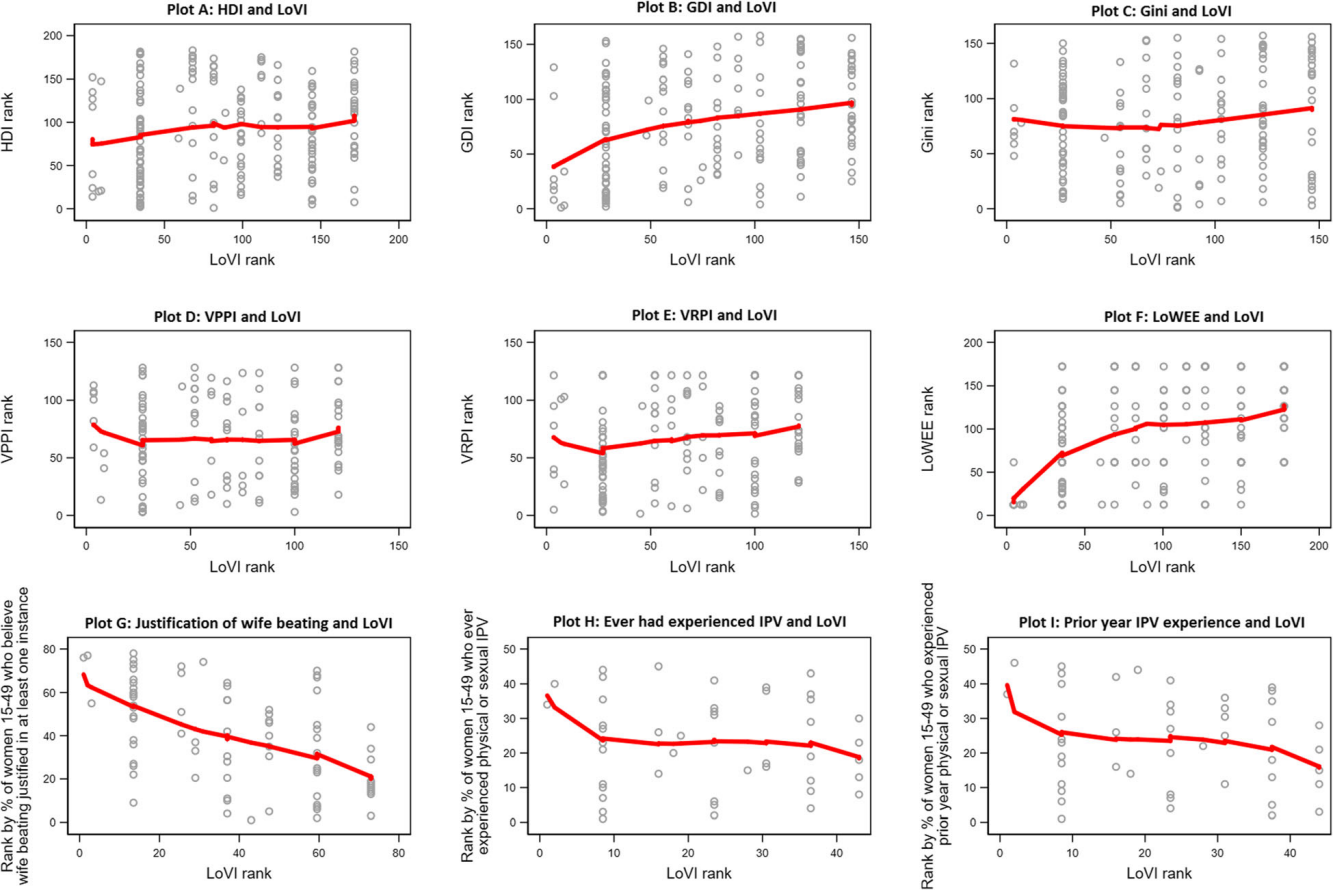
Scatterplots and LOWESS curves comparing national rankings on the LoVI with national rankings on other indicators. Notes. *HDI* Human Development Index; *GDI* Gender-related Development Index; *VPPI* Violence Against Women and Girls Prevention Program Index; *VRPI* Violence against Women and Girls Response Program Index; *LoWEE* Laws on Women’s Economic Equality index

The LOWESS regressions did not indicate a clear association of national rankings on the LoVI with national rankings on the HDI, Gini, the VAWG prevention program index (VPPI), or the VAWG response program index (VRPI) (Fig. 2). National rankings on the LoVI were positively related to national rankings on the GDI (Plot B) and the LoWEE (Plot F), suggesting that countries with more comprehensive anti-VAWG legislation tended to have achieved greater gender equality in human development and economic rights. Consistently, national rankings on the LoVI were inversely related to national rankings on the percentage of women 15–49 years who justified wife-beating in at least one instance (Plot G), who had ever experienced physical or sexual IPV (Plot H), and who had experienced prior-year physical or sexual IPV (Plot I). For experiences of IPV, this inverse relationship was strongest among countries ranking lowest and highest on the LoVI, suggesting potential threshold effects of the legal environment on IPV risk. Countries with no comprehensive legislation against VAWG had the highest percentages of women 15–49 years who justified wife beating and who experienced IPV ever and in the prior year.

**Fig. 3:**
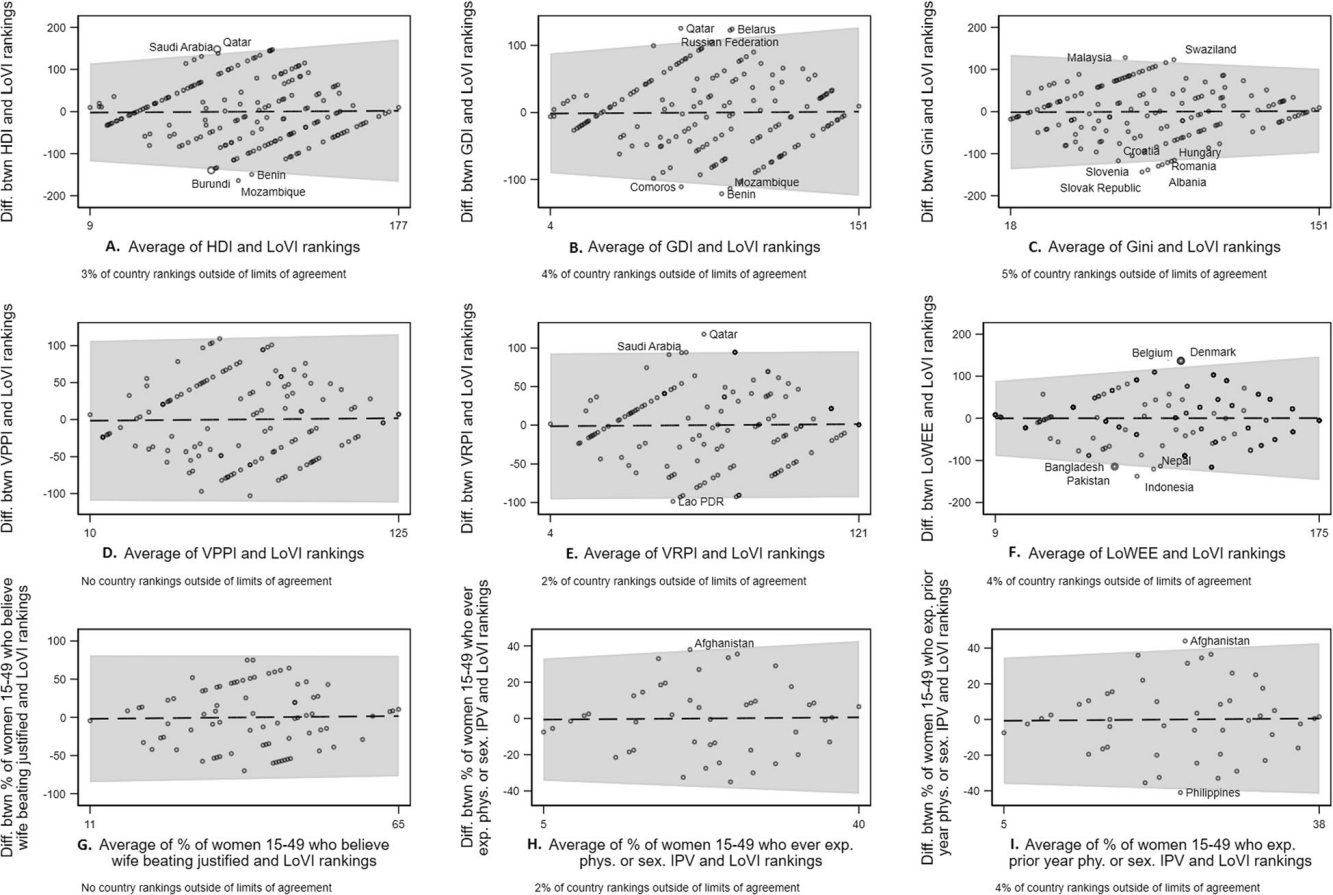
Bland-Altman plots comparing national rankings on the LoVI with national rankings on related indicators. Notes. *HDI* Human Development Index; GDI Gender-related Development Index; *VPPI* Violence against Women and Girls Prevention Program Index; *VRPI* Violence against Women and Girls Response Program Index; *LoWEE* Laws on Women’s Economic Equality index

The Bland-Altman plots (Fig. 3) indicated the level of agreement between the LoVI and related national indicators. A clustering of points along the line of equality (the dashed line at zero in Fig. 3) would have represented perfect agreement. While the LoVI rankings were not perfectly aligned with those of other national indicators, over 95% of country rankings fell within the limits of agreement (two standard deviations from the mean difference between the rankings, denoted by the gray bars in Fig. 3). Uniform scatterplots of the rankings between the lines of agreement indicate good agreement between the two measures being compared [34]. The relatively equal distribution of points above and below the line of equality indicates that LoVI rankings were not systematically higher or lower than those of other national indicators. Countries with some of the highest (Benin, Croatia, Hungary, Mozambique, Slovenia) and lowest (Qatar, Saudi Arabia) LoVI scores fell outside of the limits of agreement in comparisons between the LoVI and the HDI, GDI, Gini, and VRPI country rankings (plots A-E). When comparing the LoVI rankings to those of the HDI (plot A), GDI (plot B), LoWEE (plot F), and the percentages of women 15–49 years who had experienced lifetime (plot H) or prior-year (plot I) physical or sexual IPV, there was a greater degree of difference between rankings at higher LoVI scores. In contrast, there was a greater degree of difference between country rankings for the Gini and the LoVI (plot C) for countries with less progressive legal contexts.

## Discussion

In this article, we proposed the LoVI, a novel index measuring the comprehensiveness of national legislation against VAWG. Our descriptive summary of LoVI components for 2016 and 2018 showed some variation in national legislation on VAWG. Comprehensive national legislation against child marriage was near universal in both years, and comprehensive national legislation against sexual harassment was the least prevalent in both years. Comprehensive national legislation against sexual harassment and domestic violence was slightly more prevalent in 2018; whereas, national legislation against marital violence remained relatively less common in both years.

Regarding the prevalence of other legislation and VAWG programming, this analysis revealed substantial variation in the LoWEE, the index we created to capture national legislation with respect to women’s economic rights. National legislation supporting equality in spousal rights to inheritance was most prevalent, and gender equality in access to credit and equal pay were least prevalent. Among countries represented, response-based programming for VAWG was more widespread than programming to prevent VAWG, and reported levels of ‘full’ implementation of specific VAWG programs varied substantially (22–50% of countries ‘fully implementing’ specific prevention programs; 32–67% of countries ‘fully implementing’ specific response programs).

Regarding validation of the LoVI, factor analyses confirmed that a unidimensional index showed good model fit and was associated in expected ways with national markers of gender equality and VAWG. Importantly, countries that ranked higher on the LoVI also ranked higher on gender equality in human development and economic rights. Countries that ranked lower on the LoVI ranked higher on estimated prevalences of lifetime and prior-year IPV. However, the LoVI was not strongly associated with indices for VAWG prevention and response and programming (VPPI; VRPI). This weak association may reflect misreporting on national implementation of prevention and response programming. It also suggests that a more comprehensive legal environment does not necessarily translate into comprehensive anti-VAWG programming. Thus, in keeping with a socio-ecological model of VAWG [15], comprehensive national legislation and widespread community-based prevention and response programming are distinct and complementary strategies to address the multilevel causes of VAWG.

The LoVI was not directly comparable with other national indicators for gender equality and overall ‘development,’ but in more than 95% of cases, national rankings on the LoVI and other national indicators fell within the limits of agreement in the Bland-Altman plots of the differences between measures. This finding suggests importantly that the LoVI is a correlated, but distinct measure of the national context with respect to gender equality, women’s economic rights, and overall ‘development.’

The LoVI was created using legislative data from the World Bank. As such, the LoVI was subject to any limitations of the World Bank’s process for elucidating current laws. The World Bank does not assess the presence of laws at the national level, but rather checks for the presence of laws in major metropolitan areas. For selected countries (e.g. Nigeria), this process may mean that the laws registered in the World Bank database do not represent national laws. Given that anti-VAWG laws measured by the World Bank have changed substantially over time, the LoVI cannot be created for years prior to 2016, which was the first year that laws against marital rape and child marriage were reported in the World Bank’s WBL database. Furthermore, the VAWG indicators of laws on femicide and women trafficking are not currently collected as part of the WB-WBL. We suggest that future data collection by the World Bank use the same methodology to document the presence of and content of a broader array of VAWG laws so that the LoVI can be updated to provide an even more comprehensive measure for monitoring the legal environment related to SDG5. Finally, a few countries fell outside of the boundaries of agreement in plots of the LoVI vis-à-vis other national indicators. For all but one comparison, fewer than 5% of countries fell outside of these bounds, suggesting a generally high degree of construct validity of the LoVI vis-à-vis other measures of overall human development and of gender-related development and rights.

Despite these caveats, the LoVI is the first concise, coherent, validated index that quantifies the comprehensiveness of largely national legislation against four forms of VAWG that are globally prevalent and that occur at different stages of the life course, in different kinds of relationships, and in private and public domains of life. The LoVI’s focus on four distinct, but correlated forms of anti-VAWG legislation makes this index a critical marker of national political will to prevent VAWG. The LoVI’s generalizability is evidenced by its comparable factor structure across two calendar years and its concurrent validity with other national indicators. The LoVI, therefore, allows for comprehensive, cost-effective, and routine monitoring of the legal context regarding VAWG across countries and over time. A guide to create the LoVI is available in Supplemental Table S4.

## Conclusion

Beyond its utility to monitor SDG5, the LoVI may be used in cross-national, time-series analyses to examine the determinants of changes in national laws on VAWG, and the pathways through which anti-VAWG legislation influence’s women and girls actual experience of violence. The LoVI may be disaggregated into its component indicators to explore further the processes described above.

The LoVI is a concise, comprehensive, valid, and easy-to-estimate index for routine monitoring of national anti-VAWG legislation. Interpreting the LoVI as an index of legal norms acknowledges that national laws operate with other factors in the social ecology to influence violence against women and girls. Thus, the LoVI is a useful, complementary tool to monitor the progress of nations on advancing the 2030 SDG5, to eliminate all forms of violence against women and girls.

## Supplementary information

**Additional file 1; Supplemental Table S1.** Detailed definitions of LoVI items, from the World Bank Women Business and Law (WB-WBL) 2016 Reports and online database. **Table S2.** Distribution national laws against violence against women and girls (2016 and 2018) and other indicators (2001-2018). **Supplemental Table S3.** Laws on Violence against Women and Girls Index (LoVI) and its Component Indicators. **Supplemental Table S4.** Guide for creating the national LoVI.

## Data Availability

All data generated or analyzed in this study are included in this published article and its supplementary information files. All original data used in the analysis are national indicators or aggregates and are freely available to the public from the cited sources. To construct the Laws on VAWG Index (LoVI), data on component indicators were obtained from the World Bank, Women Business and Law (WB-WBL) database (https://wbl.worldbank.org/en/resources/data) [18, 19]. To construct the Laws on Women’s Economic Equality Index (LoWEE), data on component indicators also were obtained from the WB-WBL database (https://wbl.worldbank.org/en/resources/data) [19]. To construct the VAWG Prevention Programs Index (VPPI) and the VAWG Response Programs Index (VRPI), component indicators were obtained from the *Global Status Report on Violence 2014* [24]. To construct national prevalences for societal Norms about VAWG (justification of physical IPV, exposure to physical/sexual IPV, ever and in the prior year), data were obtained from the Demographic and Health Surveys (DHS) STATScompiler (https://www.statcompiler.com/en/) and Multiple Indicator Cluster Surveys (MICS) MICScompiler (http://www.micscompiler.org/). The Human Development Index (HDI), Gender-related Development Index (GDI), and Gini coefficient (Gini) were obtained from Human Development Report online database (http://www.hdr.undp.org/en/data).

## Abbreviations

CFA: Confirmatory Factor Analysis
CFI: Confirmatory Factor Index
DHS: Demographic and Health Surveys
EFA: Exploratory Factor Analysis
GDI: Gender-related Development Index
HDI: Human Development Index
IPV: Intimate Partner Violence
LoVI: Laws on Violence against Women and Girls Index
LoWEE: Laws on Women’s Economic Equality
RMSEA: Root mean square error of approximation
SDG5: Sustainable Development Goal 5
TLI: Tucker-Lewis Index
UN: United Nations
VAWG: Violence against women and girls
VPPI: VAWG Prevention Programs Index
VRPI: VAWG Response Programs Index
WB-WBL: World Bank Women, Business, and the Law

## Footnotes

1 The item for legal representation had a loading of 0.31; however, this item was considered theoretically salient, and was retained on those grounds. The remaining items had loadings between 0.58 and 0.92.

## References

[1] United Nations. 48/104. Declaration on the Elimination of Violence against Women: United Nations; 1993. https://www.ohchr.org/en/professionalinterest/pages/violenceagainstwomen.aspx.

[2] Clark CJ, Alonso A, Everson-Rose SA, Spencer RA, Brady SS, Resnick MD (2016). Intimate partner violence in late adolescence and young adulthood and subsequent cardiovascular risk in adulthood. Prev Med.

[3] Sigal JA, Perrino CS, Denmark FL, Dow EAA, Strashnaya R, Zarbiv T, Gielen UP, Roopnarine JL (2016). Violence against Girls. Childhood and Adolescence: Cross-Cultural Perspectives and Applications: ABC-CLIO.

[4] Solotaroff JL, Pande RP (2014). Violence against women and girls: lessons from South Asia. South Asia development forum.

[5] Yount KM, Abraham BK (2007). Female genital cutting and HIV/AIDS among Kenyan women. Stud Fam Plan.

[6] Yount KM, Crandall A, Cheong YF (2018). Women's age at first marriage and long-term economic empowerment in Egypt. World Dev.

[7] Yount KM, DiGirolamo AM, Ramakrishnan U (2011). Impacts of domestic violence on child growth and nutrition: a conceptual review of the pathways of influence. Soc Sci Med.

[8] Yount KM, Li L (2011). Domestic violence and obesity in Egyptian women. J Biosoc Sci.

[9] Yount KM, Zureick-Brown S, Salem R (2014). Intimate partner violence and Women's economic and non-economic activities in Minya, Egypt. Demography.

[10] Zureick-Brown S, Lavilla K, Yount KM (2015). Intimate partner violence and infant feeding practices in India: a cross-sectional study. Matern Child Nutr.

[11] United Nations (2015). Transforming our world: the 2030 agenda for sustainable development a/RES/70/1.

[12] Jejeebhoy SJ, Cook RJ (1997). State accountability for wife-beating: the Indian challenge. Lancet.

[13] Ellsberg M, Arango DJ, Morton M, Gennari F, Kiplesund S, Contreras M (2015). Prevention of violence against women and girls: what does the evidence say?. Lancet.

[14] Pierotti RS (2013). Increasing rejection of intimate partner violence: evidence of global cultural diffusion. Am Sociol Rev.

[15] Heise LL (1998). Violence against women: an integrated, ecological framework. Violence Against Women.

[16] Yount KM, Krause KH, Miedema SS (2017). Preventing gender-based violence victimization in adolescent girls in lower-income countries: Systematic review of reviews. Soc Sci Med.

[17] Yount KM, Crandall A, Cheong YF, Osypuk T, Naved RT, Bates LM (2016). Child marriage and intimate partner violence in rural Bangladesh: a Mulitlevel longitudinal analysis. Demography..

[18] World Bank Group (2015). Women, business, and the law 2016: getting to equal.

[19] World Bank Group (2018). Women, business, and the law 2018.

[20] Htun M, Jensenius FR, Nelson-Nuñez J (2019). Gender-discriminatory laws and women’s economic agency. Soc Polit.

[21] Hu L, Bentler PM (1999). Cutoff criteria for fit indexes in covariance structure analysis: conventional criteria versus new alternatives. Struct Equ Model Multidiscip J.

[22] Yu C-Y (2002). Evaluating cutoff criteria of model fit indices for latent variable models with binary and continuous outcomes.

[23] Sun J (2005). Assessing goodness of fit in confirmatory factor analysis. Meas Eval Couns Dev.

[24] World Health Organization (2014). United Nations Office on Drugs and Crime, United Nations Development Program. Global Status Report on Violence 2014.

[25] Demographic and Health Surveys Program (2015). Demographic and Health Surveys Model Questionnaires Demographic and Health Surveys Program.

[26] Multiple Indicator Cluster Surveys. 2018. Surveys. Retrieved from: http://mics.unicef.org/surveys.

[27] United Nations Development Program (2016). Human development report 2016: human development for everyone.

[28] Gini C. Variabilità e Mutuabilità. Contributo allo Studio delle Distribuzioni e delle Relazioni Statistiche. Bologna: C. Cuppini; 1912.

[29] Bellù LG, Liberati P (2006). Inequality analysis - the Gini index.

[30] Agresti Alan, Kateri Maria (2011). Categorical Data Analysis. International Encyclopedia of Statistical Science.

[31] Savalei V, Bonett DG, Bentler PM (2015). CFA with binary variables in small samples: a comparison of two methods. Front Psychol.

[32] Li CH (2016). Confirmatory factor analysis with ordinal data: comparing robust maximum likelihood and diagonally weighted least squares. Behav Res Methods.

[33] Bland MJ, Altman DG (1986). Statistical methods for assessing agreement between two methods of clinical measurement. Lancet.

[34] Sedgwick P (2013). Limits of agreement (Bland-Altman method). BMJ.

