# Supplementary material for: Development and validation of the LoVI: the Laws on Violence against women and girls Index
Source: BMC Int Health Hum Rights. 2020 May 29;20:13. doi: 10.1186/s12914-020-00233-z (PMC7260825; doi:10.1186/s12914-020-00233-z)
Supplement: Supplementary file 1 — Additional file 1; Supplemental Table S1. Detailed definitions of LoVI items, from the World Bank Women Business and Law (WB-WBL) 2016 Reports and online database. Table S2. Distribution national laws against violence against women and girls (2016 and 2018) and other indicators (2001-2018). Supplemental Table S3. Laws on Violence against Women and Girls Index (LoVI) and its Component Indicators. Supplemental Table S4. Guide for creating the national LoVI. [file 12914_2020_233_MOESM1_ESM.docx]

**Appendix**

**Supplemental Table S1. Detailed definitions of LoVI items, from the World Bank Women Business and Law (WB-WBL) 2016 and 2018 Reports and online database**

| **Item** | **WB-WBL definition** |
| --- | --- |
| Age at marriage | The legal age of marriage for girls is   - the age at which girls can be married without parental or another authority’s consent; or - the age under which girls are not allowed to be married, if no exceptions exist. |
| Sexual Harassment |  |
| Existence of legislation | The answer is “Yes” if   - there is a law or provision that specifically addresses and protects against sexual harassment, including unwelcome sexual advances, requests for sexual favors, verbal or physical conduct or gestures of a sexual nature, annoyance if understood to include harassment with sexual content, or any other behavior of a sexual nature that might reasonably be expected or be perceived to cause offense or humiliation to another; or - sexual harassment is considered a form of “discrimination” and legislation protects against discrimination.   The answer is “No” if   - there is no law or provision that specifically addresses sexual harassment; or - the behavior or gesture of sexual nature is performed by the use or threat of force or violence, which is understood to constitute a crime, e.g., sexual assault or abuse; or - the conduct covered in the legislation is not directed at a specific individual; or - the law only allows an employee to terminate employment based on sexual harassment but provides for no other protection or form of redress; however, a provision allowing the employer to terminate an employee’s contract for committing sexual harassment will be considered; or - the law or provision protects only a specific category of women or those in a specific area or sector of employment, e.g., protection from sexual harassment in political functions, or applicable only to government or state-owned enterprises; or - the law or provision accounts only for sexual acts, including intercourse or copulation, as clearly involving contact of or with genital organs; or - the law or provision states only that the employer has a duty to prevent sexual harassment, but no provisions exist to prohibit it or provide sanctions or other form of redress for sexual harassment; or - the law addresses harassment in general but makes no reference to acts of a sexual nature or contact. |
| Sexual harassment in employment | The question covers provisions on sexual harassment in the workplace or in employment, whether or not sanctions apply.  The answer is “Yes” if there is a law or provision that specifically protects against sexual harassment in employment, including unwelcome sexual advances, requests for sexual favors, verbal or physical conduct or gestures of a sexual nature, annoyance if understood to include harassment with sexual content, or any other behavior of a sexual nature that might reasonably be expected or be perceived to cause offense or humiliation to another in connection with employment, including provisions on inducing indecent or lewd behavior coupled with financial or official dependence or authority, abuse of position or authority, or language that can be clearly interpreted to mean such dependence or abuse; or sexual harassment is considered a form of discrimination in employment and the law protects against discrimination.  The answer is “No” if   - - there is no legislation specifically addressing sexual harassment in employment; or   - the behavior or gesture of sexual nature addressed in the law includes the use of force or violence or the threat of force or violence, which is understood to constitute a crime, e.g., sexual assault or abuse; or   - the conduct covered by legislation is not directed at a specific individual; or   - the law only allows an employee to terminate employment based on sexual harassment but provides for no other protection or form of redress; however, a provision allowing the employer to terminate an employee’s contract for committing sexual harassment will be considered; or   - the law or provision covers only public or only private sector employees, but not both; or   - the law or provision protects only a specific category of women or those in a specific area or sector of employment, e.g., protection from sexual harassment in political functions, or applicable only to government or state-owned enterprises; or   - the law accounts only for sexual acts, including intercourse or copulation, as clearly involving contact of or with genital organs; or   - the law or provision states only that the employer has a duty to prevent sexual harassment, but no provisions exist to prohibit or provide sanctions or other form of redress for sexual harassment; or   - the law addresses harassment in general but makes no reference to acts of a sexual nature or contact. |
| Sexual harassment in education | The question covers provisions addressing sexual harassment in education, education facilities, schools or where the offender is in the role of educator, professor or in charge of the education of the victim, whether or not there are sanctions associated with the provision.  The answer is “Yes” if the law specifically covers sexual harassment in education or contains language that can clearly be interpreted as sexual harassment in education.  The answer is “No” if   - - the law does not specifically cover sexual harassment in education; or   - the law covers only education within the scope of work, such as vocational training in the workplace, but not education generally; or   - the law covers sexual harassment only in public or private education, but not in both; or   - the law or provision only covers students up to a certain age (e.g., 18 years). |
| Domestic Violence |  |
| Existence of legislation | The answer is “Yes” if there is legislation addressing domestic violence, i.e., violence between spouses, within the family or members of the same household or in interpersonal relationships, including intimate partner violence that includes criminal sanctions or provides for protection orders for domestic violence.  The answer is “No” if   - - there is no legislation addressing domestic violence or the law only refers to or prohibits domestic violence but does not provide for sanctions or protection orders; or   - the law enables women to obtain a protection order only if the husband has been convicted of the offense or she has ceased to reside in the common home; or   - the law protects only a specific category of women (e.g., pregnant), a specific member of the family (e.g., children), but not all family members, or defines the offense in connection with specific motives (e.g., violence against a person while performing a professional or civil duty); or   - there is only a provision that increases penalties for general crimes covered in the criminal code if committed between spouses or within the family |
| Covers physical violence | The answer is “Yes” if   - - the term “physical” violence or abuse is clearly defined or stated in the law or provision as a form of domestic violence; or   - the law or provision includes language that can clearly be interpreted as addressing physical violence or abuse as a form of domestic violence, including cruel or inhumane treatment, or harassment if stated as affecting physical health.   The answer is “No” if   - - the law or provision does not address “physical” violence or abuse or include other language that can clearly be interpreted as physical violence or abuse as a form of domestic violence. |
| Covers sexual violence | The answer is “Yes” if   - - the term “sexual” violence or abuse is clearly defined or stated in the law or provision as a form of domestic violence; or   - the law or provision includes language that can clearly be interpreted as addressing sexual violence or abuse as a form of domestic violence.   The answer is “No” if   - - the law or provision does not address “sexual” violence or abuse or include other language that can clearly be interpreted as sexual violence or abuse as a form of domestic violence; or   - the provision on sexual abuse is not classified as occurring within the context of domestic violence or legislation on sexual abuse covers only female genital mutilation or rape |
| Covers emotional or psychological violence | The answer is “Yes” if   - - the term “psychological” or “emotional” violence or abuse is clearly defined or stated in the law or provision as a form of domestic violence; or   - the law or provision includes language that can clearly be interpreted as addressing psychological or emotional violence or abuse as a form of domestic violence, such as outrages upon personal dignity, humiliating and degrading treatment and harassment, if affecting mental health, and harm, if interpreted as emotional harm.   The answer is “No” if   - - the law or provision does not address “psychological” or “emotional” violence or abuse or include other language that can clearly be interpreted as psychological or emotional violence or abuse as a form of domestic violence. |
| Covers economic violence | The answer is “Yes” if   - - the term “financial” or “economic” violence or abuse is clearly defined or stated in the law or provision as a form of domestic violence; or   - the law or provision includes language that can clearly be interpreted as addressing financial or economic violence or abuse as a form of domestic violence, such as deprivation or limitation of the spouse’s access to goods and financial resources; or   - the use of violence that causes or results in consequences affecting the livelihood of the victim.   The answer is “No” if   - - the law or provision does not address “financial” or “economic” violence or abuse, or include other language that can clearly be interpreted as financial or economic violence or abuse; or   - covers only the use of violence that causes the destruction of property. |
| Criminalization of marital rape | The answer is “Yes” if   - - there is legislation that explicitly criminalizes the act of marital rape by providing that rape or sexual assault provisions apply irrespective of the nature of the relationship between the perpetrator and victim, or that no marriage or other relationship shall constitute a defense to a charge of rape or sexual assault under the legislation; or   - there is legislation that explicitly criminalizes the act of rape between (1) persons in marital relationships; (2) relatives, when the law explicitly recognizes spouses as  relatives (but not for relatives in general); or (3) persons in situations of abuse or dependency of family, when the law clearly includes spouses within the definition of family; or when legislation that explicitly criminalizes the act of rape states that the spouse is a potential offender or is not exempt from charges; or   - marital relationships are an aggravating factor for the crimes of rape or sexual assault that includes elements of rape, or if the law sets out conditions in which the penalty for marital rape or rape by the husband is mitigated so that the criminalization of marital rape can be inferred.   The answer is “No” if   - - there are no criminal sanctions for the offense of rape between spouses (i.e., the law only “prohibits” the act, provides for the application of protection orders or allows a judge to order a husband not to rape his wife); or   - the provision on marital rape applies only if the spouses are separated or in the process of getting separated; or   - the provision covers only relationships of dependency in general, or financial or official dependence; or   - the provision on rape applies only in certain circumstances, such as sickness or pregnancy; or   - the provision applies only to family members, but spouses are not clearly included in the definition of family; or   - the provision on marital rape states that prosecution may only be instituted with authorization of the Attorney General or an authority with discretionary powers. |

Note. This material comes from *Women, Business, and the Law 2016: Getting to Equal* and *Women, Business, and the Law 2018* (18, 19). This work is available for distribution under the Creative Commons Attribution 3.0 IGO license (CC BY 3.0 IGO) http://creativecommons.org/ licenses/by/3.0/igo. The full sources are, below, and appear in the references in the manuscript.

Sources:

World Bank Group. 2015. Women, Business and the Law 2016: Getting to Equal. Washington, DC: World Bank. doi:10.1596/978-1-4648-0677-3. License: Creative Commons Attribution CC BY 3.0 IGO

World Bank Group. 2018. Women, Business and the Law 2018. Washington, DC: World Bank. License: Creative Commons Attribution CC BY 3.0 IGO.

**Table S2. Distribution national laws against violence against women and girls (2016 and 2018) and other indicators (2001-2018)**

| **National laws on VAWG**^a^ | **2016** | | **2018** | |  |  |  |  |
| --- | --- | --- | --- | --- | --- | --- | --- | --- |
|  | **N** | **(%)** | **N** | **(%)** |  |  |  |  |
| Legal minimum age of marriage for girls is 18+ (N=186)^b^ | 174 | (93.6) | 174 | (93.6) |  |  |  |  |
| ***Legislation on sexual harassment*** (N=189) | 54 | (28.6) | 64 | (33.9) |  |  |  |  |
| Existence of sexual harassment legislation | 144 | (76.2) | 154 | (81.5) |  |  |  |  |
| Legislation covers sexual harassment in employment | 119 | (63.0) | 130 | (68.8) |  |  |  |  |
| Legislation covers sexual harassment in education | 56 | (29.6) | 66 | (34.9) |  |  |  |  |
| ***Legislation on domestic violence*** (N=189) | 82 | (43.4) | 93 | (49.2) |  |  |  |  |
| Existence of domestic violence legislation | 140 | (74.1) | 144 | (76.2) |  |  |  |  |
| Legislation covers physical violence | 137 | (72.5) | 142 | 75.1) |  |  |  |  |
| Legislation covers sexual violence | 106 | (56.1) | 119 | (63.0) |  |  |  |  |
| Legislation covers emotional violence | 134 | (70.9) | 141 | (74.6) |  |  |  |  |
| Legislation covers economic violence | 86 | (45.5) | 95 | (50.3) |  |  |  |  |
| Legislation explicitly criminalizes marital rape (N=189) | 76 | (40.2) | 78 | (41.3) |  |  |  |  |
|  |  |  | **2018** | |  |  |  |  |
| ***Laws on women’s economic equality 2018***^a^ |  |  | **N** | **(%)** |  |  |  |  |
| The law mandates nondiscrimination based on gender in employment & hiring (N=189) |  |  | 95 | (50.3) |  |  |  |  |
| The law mandates equal remuneration for work of equal value (N=189) |  |  | 76 | (40.2) |  |  |  |  |
| Sons and daughters have equal rights to inherit assets from their parents (N=185) |  |  | 146 | (78.9) |  |  |  |  |
| Female and male surviving spouses have equal rights to inherit assets (N=183) |  |  | 147 | (80.3) |  |  |  |  |
| The law prohibits discrimination based sex or gender in access to credit (N=189) |  |  | 72 | (38.1) |  |  |  |  |
| ***VAWG prevention and response programs***^c^ | **None** | | **Limited** | | **Full** | |  |  |
| ***VAWG prevention programs*** | **N** | **(%)** | **N** | **(%)** | **N** | **(%)** |  |  |
| Socio-cultural norms change related to sexual violence (N=133) | 14 | (10.5) | 52 | (39.1) | 67 | (50.4) |  |  |
| Socio-cultural norms change related to IPV (N=131) | 13 | (9.9) | 53 | (40.5) | 65 | (49.6) |  |  |
| Sexual violence prevention in schools or colleges (N=131) | 24 | (18.3) | 59 | (45.0) | 48 | (36.6) |  |  |
| Changes to the environment to prevent sexual violence (N=131) | 32 | (24.4) | 60 | (45.8) | 39 | (29.8) |  |  |
| Dating violence prevention in schools (N=129) | 58 | (45.0) | 42 | (32.6) | 29 | (22.5) |  |  |
| Microfinance and gender equity training (N=130) | 54 | (41.5) | 48 | (36.9) | 28 | (21.5) |  |  |
| ***VAWG response programs*** |  |  |  |  |  |  |  |  |
| Medico-legal services for sexual violence survivors (N=132) | 8 | (6.1) | 35 | (26.5) | 89 | (67.4) |  |  |
| Health-provider identification and referral of IPV survivors (N=132) | 18 | (13.6) | 43 | (32.6) | 71 | (53.8) |  |  |
| Prenatal screening for child maltreatment and IPV risks (N=132) | 39 | (29.6) | 41 | (31.1) | 52 | (39.4) |  |  |
|  | **None** | | **Limited** | | **Partial** | | **Full** | |
|  | **N** | **(%)** | **N** | **(%)** | **N** | **(%)** | **N** | **(%)** |
| Victim representation (N=130) | 15 | (11.5) | 20 | (15.4) | 25 | (19.2) | 70 | (53.9) |
| Victim compensation (N=130) | 60 | (46.2) | 14 | (10.8) | 15 | (11.5) | 41 | (31.5) |
| ***Societal norms about VAWG*** | **Mean** | **(SD)** | **Range** |  |  |  |  |  |
| % of women 15-49 who believe wife beating justified in at least one instance (N=78)^d^ | 36.3 | (22.1) | 2.3-92.1 |  |  |  |  |  |
| % of women 15-49 who ever experienced physical or sexual violence (N=45)^e^ | 34.2 | (13.0) | 5.9-62.5 |  |  |  |  |  |
| % of women 15-49 who experienced prior year physical or sexual IPV (N=46)^e^ | 19.0 | (9.4) | 3.5-46.1 |  |  |  |  |  |
| ***Human development and income inequality***^f^ |  |  |  |  |  |  |  |  |
| Human Development Index (HDI), range 0-1 (N=183) | 0.7 | (0.2) | 0.4-0.9 |  |  |  |  |  |
| Gender-Related Human Development Index (GDI), range 0-2 (N=158) | 0.9 | (0.1) | 0.6-1.0 |  |  |  |  |  |
| Gini, range 0-100 (N=157) | 38.1 | (8.1) | 16.6-63 |  |  |  |  |  |

*SD* standard deviation; *VAWG* violence against women and girls

^a^ World Bank (WB) Women, Business and Law (WBL) database. These data are freely available to the public at the following website: <https://wbl.worldbank.org/en/resources/data>.

^b^ In 2016, three countries had missing data on legal age at marriage for girls (Lebanon, Saudi Arabia, Republic of Yemen). In 2018, 19 countries had missing data on the legal age at marriage for girls. For 16 of these 19 countries, missing data were imputed using the status of the law in 2016, resulting in three countries with missing data in 2018 (Lebanon, Saudi Arabia, Republic of Yemen).

^c^ World Health Organization, United Nations Office on Drugs and Crime, United Nations Development Program. Global Status Report on Violence 2014. Geneva, CH: World Health Organization; 2014.

^d^ The Demographic and Health Surveys (DHS) STATScompiler (<https://www.statcompiler.com/en/>) and Multiple Indicator Cluster Surveys (MICS) MICScompiler (<http://www.micscompiler.org/>) provide these data freely to the public.

^e^ DHS STATCOMPILER (<https://www.statcompiler.com/en/>) freely available to the public.

^f^ United Nations Development Program. Human Development Report online database (<http://www.hdr.undp.org/en/data>).

**Supplemental Table S3. Laws on Violence against Women and Girls Index (LoVI) and its Component Indicators**

| LoVI Rank | Country | LoVI | Comprehensive Sexual Harassment Legislation | Legal Age of Marriage in Years | Comprehensive Domestic Violence Legislation | Marital Rape Criminalized |
| --- | --- | --- | --- | --- | --- | --- |
| 1 | Albania | 0.87 | Yes | 18 or older | Yes | Yes |
| 1 | Australia | 0.87 | Yes | 18 or older | Yes | Yes |
| 1 | Belize | 0.87 | Yes | 18 or older | Yes | Yes |
| 1 | Benin | 0.87 | Yes | 18 or older | Yes | Yes |
| 1 | Bolivia | 0.87 | Yes | 18 or older | Yes | Yes |
| 1 | Costa Rica | 0.87 | Yes | 18 or older | Yes | Yes |
| 1 | Croatia | 0.87 | Yes | 18 or older | Yes | Yes |
| 1 | Honduras | 0.87 | Yes | 18 or older | Yes | Yes |
| 1 | Hungary | 0.87 | Yes | 18 or older | Yes | Yes |
| 1 | Korea, Rep. | 0.87 | Yes | 18 or older | Yes | Yes |
| 1 | Mauritius | 0.87 | Yes | 18 or older | Yes | Yes |
| 1 | Mexico | 0.87 | Yes | 18 or older | Yes | Yes |
| 1 | Mozambique | 0.87 | Yes | 18 or older | Yes | Yes |
| 1 | Namibia | 0.87 | Yes | 18 or older | Yes | Yes |
| 1 | New Zealand | 0.87 | Yes | 18 or older | Yes | Yes |
| 1 | Nicaragua | 0.87 | Yes | 18 or older | Yes | Yes |
| 1 | Panama | 0.87 | Yes | 18 or older | Yes | Yes |
| 1 | Peru | 0.87 | Yes | 18 or older | Yes | Yes |
| 1 | Philippines | 0.87 | Yes | 18 or older | Yes | Yes |
| 1 | Romania | 0.87 | Yes | 18 or older | Yes | Yes |
| 1 | Slovak Republic | 0.87 | Yes | 18 or older | Yes | Yes |
| 1 | Slovenia | 0.87 | Yes | 18 or older | Yes | Yes |
| 1 | Turkey | 0.87 | Yes | 18 or older | Yes | Yes |
| 1 | Venezuela, RB | 0.87 | Yes | 18 or older | Yes | Yes |
| 2 | Argentina | 0.76 | No | 18 or older | Yes | Yes |
| 2 | Barbados | 0.76 | No | 18 or older | Yes | Yes |
| 2 | Bhutan | 0.76 | No | 18 or older | Yes | Yes |
| 2 | Brazil | 0.76 | No | 18 or older | Yes | Yes |
| 2 | Burundi | 0.76 | No | 18 or older | Yes | Yes |
| 2 | Cabo Verde | 0.76 | No | 18 or older | Yes | Yes |
| 2 | Colombia | 0.76 | No | 18 or older | Yes | Yes |
| 2 | Comoros | 0.76 | No | 18 or older | Yes | Yes |
| 2 | Dominica | 0.76 | No | 18 or older | Yes | Yes |
| 2 | Georgia | 0.76 | No | 18 or older | Yes | Yes |
| 2 | Grenada | 0.76 | No | 18 or older | Yes | Yes |
| 2 | Guatemala | 0.76 | No | 18 or older | Yes | Yes |
| 2 | Guinea-Bissau | 0.76 | No | 18 or older | Yes | Yes |
| 2 | Indonesia | 0.76 | No | 18 or older | Yes | Yes |
| 2 | Italy | 0.76 | No | 18 or older | Yes | Yes |
| 2 | Lao PDR | 0.76 | No | 18 or older | Yes | Yes |
| 2 | Latvia | 0.76 | No | 18 or older | Yes | Yes |
| 2 | Moldova | 0.76 | No | 18 or older | Yes | Yes |
| 2 | Mongolia | 0.76 | No | 18 or older | Yes | Yes |
| 2 | Nepal | 0.76 | No | 18 or older | Yes | Yes |
| 2 | Portugal | 0.76 | No | 18 or older | Yes | Yes |
| 2 | Rwanda | 0.76 | No | 18 or older | Yes | Yes |
| 2 | San MariNo | 0.76 | No | 18 or older | Yes | Yes |
| 2 | São Tomé and Príncipe | 0.76 | No | 18 or older | Yes | Yes |
| 2 | Serbia | 0.76 | No | 18 or older | Yes | Yes |
| 2 | Sierra Leone | 0.76 | No | 18 or older | Yes | Yes |
| 2 | Solomon Islands | 0.76 | No | 18 or older | Yes | Yes |
| 2 | South Africa | 0.76 | No | 18 or older | Yes | Yes |
| 2 | Suriname | 0.76 | No | 18 or older | Yes | Yes |
| 2 | Trinidad and Tobago | 0.76 | No | 18 or older | Yes | Yes |
| 2 | Vietnam | 0.76 | No | 18 or older | Yes | Yes |
| 3 | Bangladesh | 0.63 | Yes | 18 or older | Yes | No |
| 3 | Bosnia and Herzegovina | 0.63 | Yes | 18 or older | Yes | No |
| 3 | Bulgaria | 0.63 | Yes | 18 or older | Yes | No |
| 3 | El Salvador | 0.63 | Yes | 18 or older | Yes | No |
| 3 | India | 0.63 | Yes | 18 or older | Yes | No |
| 3 | Israel | 0.63 | Yes | 18 or older | Yes | No |
| 3 | Kenya | 0.63 | Yes | 18 or older | Yes | No |
| 3 | Kosovo | 0.63 | Yes | 18 or older | Yes | No |
| 3 | Lithuania | 0.63 | Yes | 18 or older | Yes | No |
| 3 | Macedonia, FYR | 0.63 | Yes | 18 or older | Yes | No |
| 3 | Maldives | 0.63 | Yes | 18 or older | Yes | No |
| 3 | Nigeria | 0.63 | Yes | 18 or older | Yes | No |
| 3 | Pakistan | 0.63 | Yes | 18 or older | Yes | No |
| 3 | Uruguay | 0.63 | Yes | 18 or older | Yes | No |
| 3 | Zambia | 0.63 | Yes | 18 or older | Yes | No |
| 4 | Dominican Republic | 0.56 | Yes | 18 or older | No | Yes |
| 4 | Ecuador | 0.56 | Yes | 18 or older | No | Yes |
| 4 | Estonia | 0.56 | Yes | 18 or older | No | Yes |
| 4 | Hong Kong SAR, China | 0.56 | Yes | 18 or older | No | Yes |
| 4 | Iceland | 0.56 | Yes | 18 or older | No | Yes |
| 4 | Malta | 0.56 | Yes | 18 or older | No | Yes |
| 4 | Puerto Rico (U.S.) | 0.56 | Yes | 18 or older | No | Yes |
| 4 | Sweden | 0.56 | Yes | 18 or older | No | Yes |
| 4 | Taiwan, China | 0.56 | Yes | 18 or older | No | Yes |
| 5 | Angola | 0.54 | No | 18 or older | Yes | No |
| 5 | Antigua and Barbuda | 0.54 | No | 18 or older | Yes | No |
| 5 | Azerbaijan | 0.54 | No | 18 or older | Yes | No |
| 5 | Bahamas, The | 0.54 | No | 18 or older | Yes | No |
| 5 | Botswana | 0.54 | No | 18 or older | Yes | No |
| 5 | Gambia, The | 0.54 | No | 18 or older | Yes | No |
| 5 | Ghana | 0.54 | No | 18 or older | Yes | No |
| 5 | Kazakhstan | 0.54 | No | 18 or older | Yes | No |
| 5 | Kiribati | 0.54 | No | 18 or older | Yes | No |
| 5 | Malawi | 0.54 | No | 18 or older | Yes | No |
| 5 | Marshall Islands | 0.54 | No | 18 or older | Yes | No |
| 5 | Montenegro | 0.54 | No | 18 or older | Yes | No |
| 5 | Paraguay | 0.54 | No | 18 or older | Yes | No |
| 5 | St. Kitts and Nevis | 0.54 | No | 18 or older | Yes | No |
| 5 | St. Vincent and the Grenadines | 0.54 | No | 18 or older | Yes | No |
| 5 | Tajikistan | 0.54 | No | 18 or older | Yes | No |
| 5 | Tonga | 0.54 | No | 18 or older | Yes | No |
| 5 | Uganda | 0.54 | No | 18 or older | Yes | No |
| 5 | Ukraine | 0.54 | No | 18 or older | Yes | No |
| 5 | Zimbabwe | 0.54 | No | 18 or older | Yes | No |
| 6 | Lebanon | 0.53 | No | Missing | Yes | No |
| 7 | Timor-Leste | 0.51 | No | Below 18 | Yes | Yes |
| 8 | Canada | 0.48 | No | 18 or older | No | Yes |
| 8 | Central African Republic | 0.48 | No | 18 or older | No | Yes |
| 8 | Chile | 0.48 | No | 18 or older | No | Yes |
| 8 | Cyprus | 0.48 | No | 18 or older | No | Yes |
| 8 | France | 0.48 | No | 18 or older | No | Yes |
| 8 | Greece | 0.48 | No | 18 or older | No | Yes |
| 8 | Guyana | 0.48 | No | 18 or older | No | Yes |
| 8 | Lesotho | 0.48 | No | 18 or older | No | Yes |
| 8 | Luxembourg | 0.48 | No | 18 or older | No | Yes |
| 8 | Samoa | 0.48 | No | 18 or older | No | Yes |
| 8 | Thailand | 0.48 | No | 18 or older | No | Yes |
| 8 | Togo | 0.48 | No | 18 or older | No | Yes |
| 9 | Cameroon | 0.35 | Yes | 18 or older | No | No |
| 9 | Czech Republic | 0.35 | Yes | 18 or older | No | No |
| 9 | Egypt, Arab Rep. | 0.35 | Yes | 18 or older | No | No |
| 9 | Eritrea | 0.35 | Yes | 18 or older | No | No |
| 9 | Ethiopia | 0.35 | Yes | 18 or older | No | No |
| 9 | Fiji | 0.35 | Yes | 18 or older | No | No |
| 9 | Finland | 0.35 | Yes | 18 or older | No | No |
| 9 | Ireland | 0.35 | Yes | 18 or older | No | No |
| 9 | Netherlands | 0.35 | Yes | 18 or older | No | No |
| 9 | Norway | 0.35 | Yes | 18 or older | No | No |
| 9 | Poland | 0.35 | Yes | 18 or older | No | No |
| 9 | Spain | 0.35 | Yes | 18 or older | No | No |
| 9 | Sri Lanka | 0.35 | Yes | 18 or older | No | No |
| 9 | United Kingdom | 0.35 | Yes | 18 or older | No | No |
| 9 | United States | 0.35 | Yes | 18 or older | No | No |
| 10 | Bahrain | 0.33 | No | Below 18 | Yes | No |
| 11 | Uzbekistan | 0.27 | No | Below 18 | No | Yes |
| 12 | Algeria | 0.24 | No | 18 or older | No | No |
| 12 | Armenia | 0.24 | No | 18 or older | No | No |
| 12 | Austria | 0.24 | No | 18 or older | No | No |
| 12 | Belarus | 0.24 | No | 18 or older | No | No |
| 12 | Belgium | 0.24 | No | 18 or older | No | No |
| 12 | Brunei Darussalam | 0.24 | No | 18 or older | No | No |
| 12 | Burkina Faso | 0.24 | No | 18 or older | No | No |
| 12 | Cambodia | 0.24 | No | 18 or older | No | No |
| 12 | Chad | 0.24 | No | 18 or older | No | No |
| 12 | China | 0.24 | No | 18 or older | No | No |
| 12 | Congo, Dem. Rep. | 0.24 | No | 18 or older | No | No |
| 12 | Congo, Rep. | 0.24 | No | 18 or older | No | No |
| 12 | Côte d'Ivoire | 0.24 | No | 18 or older | No | No |
| 12 | Denmark | 0.24 | No | 18 or older | No | No |
| 12 | Djibouti | 0.24 | No | 18 or older | No | No |
| 12 | Equatorial Guinea | 0.24 | No | 18 or older | No | No |
| 12 | Gabon | 0.24 | No | 18 or older | No | No |
| 12 | Germany | 0.24 | No | 18 or older | No | No |
| 12 | Guinea | 0.24 | No | 18 or older | No | No |
| 12 | Haiti | 0.24 | No | 18 or older | No | No |
| 12 | Iraq | 0.24 | No | 18 or older | No | No |
| 12 | Jamaica | 0.24 | No | 18 or older | No | No |
| 12 | Japan | 0.24 | No | 18 or older | No | No |
| 12 | Jordan | 0.24 | No | 18 or older | No | No |
| 12 | Kyrgyz Republic | 0.24 | No | 18 or older | No | No |
| 12 | Liberia | 0.24 | No | 18 or older | No | No |
| 12 | Libya | 0.24 | No | 18 or older | No | No |
| 12 | Madagascar | 0.24 | No | 18 or older | No | No |
| 12 | Mauritania | 0.24 | No | 18 or older | No | No |
| 12 | Micronesia, Fed. Sts. | 0.24 | No | 18 or older | No | No |
| 12 | Morocco | 0.24 | No | 18 or older | No | No |
| 12 | Myanmar | 0.24 | No | 18 or older | No | No |
| 12 | Niger | 0.24 | No | 18 or older | No | No |
| 12 | Oman | 0.24 | No | 18 or older | No | No |
| 12 | Palau | 0.24 | No | 18 or older | No | No |
| 12 | Papua New Guinea | 0.24 | No | 18 or older | No | No |
| 12 | Russian Federation | 0.24 | No | 18 or older | No | No |
| 12 | Senegal | 0.24 | No | 18 or older | No | No |
| 12 | Seychelles | 0.24 | No | 18 or older | No | No |
| 12 | Singapore | 0.24 | No | 18 or older | No | No |
| 12 | South Sudan | 0.24 | No | 18 or older | No | No |
| 12 | St. Lucia | 0.24 | No | 18 or older | No | No |
| 12 | Swaziland | 0.24 | No | 18 or older | No | No |
| 12 | Switzerland | 0.24 | No | 18 or older | No | No |
| 12 | Tanzania | 0.24 | No | 18 or older | No | No |
| 12 | Tunisia | 0.24 | No | 18 or older | No | No |
| 12 | United Arab Emirates | 0.24 | No | 18 or older | No | No |
| 12 | Vanuatu | 0.24 | No | 18 or older | No | No |
| 13 | Saudi Arabia | 0.22 | No | Missing | No | No |
| 13 | Yemen, Rep. | 0.22 | No | Missing | No | No |
| 14 | Afghanistan | 0.12 | Yes | Below 18 | No | No |
| 15 | Iran, Islamic Rep. | 0.00 | No | Below 18 | No | No |
| 15 | Kuwait | 0.00 | No | Below 18 | No | No |
| 15 | Malaysia | 0.00 | No | Below 18 | No | No |
| 15 | Mali | 0.00 | No | Below 18 | No | No |
| 15 | Qatar | 0.00 | No | Below 18 | No | No |
| 15 | Sudan | 0.00 | No | Below 18 | No | No |
| 15 | Syrian Arab Republic | 0.00 | No | Below 18 | No | No |
| 15 | West Bank and Gaza | 0.00 | No | Below 18 | No | No |
|  |  |  |  |  |  |  |
| Rank by Region | |  |  |  |  |  |
| Rank | **Regions** | **Average LoVI** |  |  |  |  |
| 1 | Latin America & Caribbean | 0.67 |  |  |  |  |
| 2 | Europe & Central Asia | 0.59 |  |  |  |  |
| 3 | South Asia | 0.56 |  |  |  |  |
| 4 | High Income: OECD | 0.50 |  |  |  |  |
| 5 | Sub-Saharan Africa | 0.46 |  |  |  |  |
| 6 | East Asia & Pacific | 0.46 |  |  |  |  |
| 7 | Middle East & North Africa | 0.22 |  |  |  |  |
|  |  |  |  |  |  |  |
| Rank by Income Group | |  |  |  |  |  |
| Rank | **Income Group** | **Average LoVI** |  |  |  |  |
| 1 | Upper middle income | 0.57 |  |  |  |  |
| 2 | Lower middle income | 0.49 |  |  |  |  |
| 3 | High Income | 0.48 |  |  |  |  |
| 4 | Low Income | 0.44 |  |  |  |  |
| Original data from which these indicators and the LoVI are derived are freely available from the WB-WBL World Bank, Women Business and Law database: <https://wbl.worldbank.org/en/resources/data> | | | | | | |

**Supplemental Table S4. Guide for creating the national LoVI**

| 1. **Access the World Bank – Women Business and Law data source** | | | |
| --- | --- | --- | --- |
| The World Bank: Women, Business, and the Law <http://wbl.worldbank.org/>  Protecting Women from Violence: <http://wbl.worldbank.org/en/methodology#a>  WB-WBL World Bank, Women Business and Law database: <https://wbl.worldbank.org/en/resources/data> | | | |
| 1. **Recode the original items** | | **Original Code** | **Variable Recode** |
| *Child Marriage (N=186):* | |  |  |
| What is the legal age of marriage for girls? | | Years (Range 0-25) | <18=0; ≥18=1 |
| *Sexual Harassment (N=189):* | |  |  |
| Is there legislation that specifically addresses sexual harassment? | | Yes/No | Yes=1; No=0 |
| Is there legislation on sexual harassment in employment? | | Yes/No | Yes=1; No=0 |
| Is there legislation on sexual harassment in education? | | Yes/No | Yes=1; No=0 |
| *Domestic Violence (N=189):* | |  |  |
| Is there domestic violence legislation? | | Yes/No | Yes=1; No=0 |
| Does domestic violence legislation cover physical violence? | | Yes/No | Yes=1; No=0 |
| Does domestic violence legislation cover sexual violence? | | Yes/No | Yes=1; No=0 |
| Does domestic violence legislation cover emotional/psychological violence? | | Yes/No | Yes=1; No=0 |
| Does domestic violence legislation cover economic or financial violence? | | Yes/No | Yes=1; No=0 |
| *Marital Rape (N=189):* | |  |  |
| Does legislation explicitly criminalize marital rape? | | Yes/No | Yes=1; No=0 |
| 1. **Code the composite indicators** | | | |
| Domestic Violence (DV) Composite=1 if all 5 DV items=Yes, otherwise DV composite=0 | | | |
| Sexual Harassment (SH) Composite=1 if all 3 SH items=Yes, otherwise SH composite=0 | | | |
| 1. **Use factor loadings from the confirmatory factor analysis to create the initial LoVI index** | | | |
| LoVI Composite Indicator | WB 2018 CFA | |  |
| 1. Child marriage 2. Sexual harassment legislation 3. Domestic violence legislation 4. Marital rape legislation 5. Examples: 6. Albania: 0.70*1+0.39*1+0.79*1+0.61*1 7. Ghana: 0.70*1+0.39*0+0.79*1+0.61*0 8. Syrian Arab Rep: 0.70*0+0.39*0+0.79*0+0.61*0 | 0.70  0.39  0.79  0.61 | |  |
| 1. **Re-scale the confirmatory factor scores** | | | |
| 1. Standardize the index by subtracting the mean from the original score and dividing by the standard deviation: x* = (x-m)/sd 2. Rescale the index to 0 to 1 by rounding all values greater than 2 to 2 and rounding all values less than -2 to -2. Divide all values by 4 to obtain a 1-point range with a mean of 0. 3. Add 0.5 to all values to rescale the index to a mean of 0.5 and a range from 0 to 1. | | | |
| 1. **Check your work with illustrative LoVI scores** | | | |
| 1. Highest ranked country: Albania: 0.8725 2. Median ranked country: Ghana: 0.5384 3. Lowest ranked country: Syrian Arab Republic: 0.0000 | | | |
